# Development of a high-throughput method to screen novel antiviral materials

**DOI:** 10.1371/journal.pone.0266474

**Published:** 2022-04-27

**Authors:** Makoto Nakakido, Naoki Tanaka, Ayako Shimojo, Nobuhiro Miyamae, Kouhei Tsumoto

**Affiliations:** 1 Department of Bioengineering, School of Engineering, The University of Tokyo, Bunkyo-ku, Tokyo, Japan; 2 Department of Chemistry & Biotechnology, School of Engineering, The University of Tokyo, Bunkyo-ku, Tokyo, Japan; 3 Nippon Paint Co., LTD, Shinagawa-ku, Tokyo, Japan; 4 Medical Proteomics Laboratory, The Institute of Medical Science, The University of Tokyo, Minato-ku, Tokyo, Japan; Argonne National Laboratory, UNITED STATES

## Abstract

Respiratory infectious diseases pose a serious threat worldwide, and novel antiviral materials are highly demanded. Photocatalytic nanoparticles have been developed to inhibit indirect transmission of pathogens by acting as surface coating materials. During development of such antiviral materials, researchers use bacteriophages as model viruses due to their safety and experimental efficiency. Screening methods are used to identify potential antiviral materials, and better screening technologies will accelerate the discovery of antiviral treatments. In this study, we constructed a novel platform to evaluate antiviral activity of surface coating materials using the M13 bacteriophage and phagemid system derived from phage display technology. The evaluation results generated by this system for the two tested antiviral materials were comparable to those for the materials tested on the Qβ bacteriophage and influenza virus using traditional screening methods. The experimental system developed in this study provides rapid and effective screening and can be applied to the development of novel antiviral materials.

## Introduction

Infectious diseases pose a serious threat worldwide, and respiratory viruses in particular can cause rapid and explosive infections. Influenza viruses were responsible for global pandemics in 1918, 1957, 1968, and 2009 [1, 2]. More recently, coronavirus 2 caused the COVID-19 global pandemic, which resulted in more than 6.0 million deaths and 448 million cases as of 10 March 2022 [3]. In addition to human health impacts, pandemics have significant negative economic and social impacts due to measures introduced to reduce the spread of infection. Therefore, developing methods to suppress respiratory infections requires urgent attention.

Respiratory viruses can be transmitted via direct contact, indirect contact, droplets, or aerosols [4], and a variety of nanotechnology-based materials have been developed to inhibit each transmission pathway [5]. Among them, photocatalytic nanoparticles such as titanium dioxide (TiO_2_)-based photocatalysts have been used for surface disinfection, resulting in inhibition of indirect transmission [6, 7]. In particular, Cu(II) nanocluster-grafted TiO_2_ has been shown to effectively inactivate viruses under visible light and is now being used as an antiviral surface coating material in indoor environments [8, 9].

During development of antiviral materials, bacteriophages are often used to evaluate antiviral activity. Bacteriophages specifically infect bacteria and archaea and therefore do not pose the risk of human infection [9–11]. In addition, high-throughput techniques can be used in bacteriophage-based experiments because bacteria grow more rapidly than mammalian cells, thus the evaluation process is faster and more effective. Further improvements in experimental efficiency will accelerate the development of novel antiviral materials.

**Fig 1:**
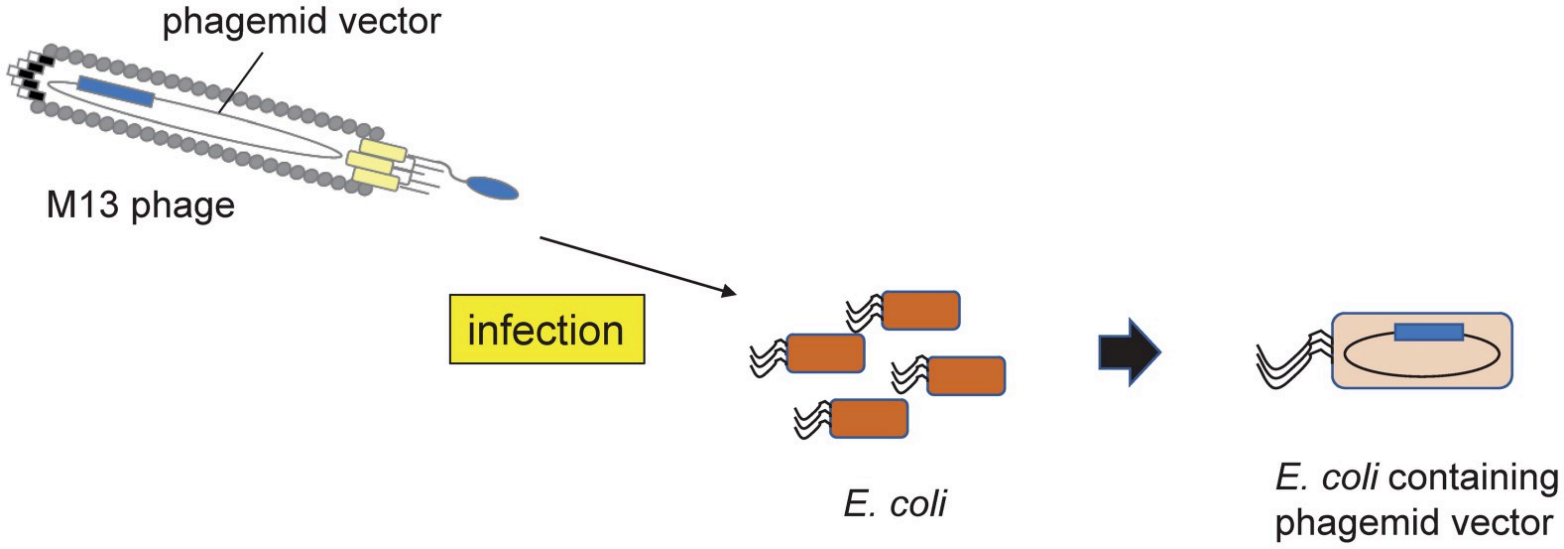
Schematic figure showing introduction of the phagemid upon infection. *E*. *coli* infected by M13 phages acquire antibiotic resistance derived from the phagemid vector.

Phage display technology enable peptides and proteins to be displayed on the surface of bacteriophages [12, 13], and it is a robust platform for screening antibody fragments as antigen binders [14–16]. Although a variety of bacteriophages have been used in this system, the M13 filamentous phage is the one most commonly utilized [17, 18]. In the system, a phagemid vector containing the genes for the displayed proteins is introduced into *Escherichia coli* (*E*.*coli*) upon infection, and the vector provides antibiotic resistance to the *E*.*coli* cells, thereby allowing the antibiotic-based selection (Fig 1).

In this study, we constructed a novel platform to evaluate the antiviral activity of materials using the M13 bacteriophage and phagemid system derived from the phage display system. *E*. *coli* cells were infected with the M13 bacteriophage treated with antiviral materials, and active M13 phages were evaluated by colony counting. Our platform enables more effective screening than previous bacteriophage-based evaluation systems that rely on plaque formation assays, and it should be applied to the development of novel antiviral materials.

## Materials and methods

### Preparation of M13 bacteriophages

Phage stocks were prepared as described previously [19]. Briefly, the *E*. *coli* stock containing an antibody library was grown in broth and infected with helper phages. After overnight culture, phages were purified by polyethylene glycol precipitation and dissolved in SM buffer (10 mM Tris-HCl pH 7.5 100 mM NaCl 8 mM MgSO_4_). Purified phages were stored at 4°C.

### Coating of glass slides with anti-viral materials

Glass slides coated with each anti-viral material were prepared as follows. The anti-viral materials were dropped onto the glass slide using a dropper. The PROTECTON BARRIERX Spray was coated using Bar corter No.4 (AXEL, Japan) and the PROTECTON INTERIORWALL VK-500 was coated using an applicator with 10 mil standard film thickness (TP-giken, Japan). The coated glass was dried for 24 hours (PROTECTON BARRIERX Spray) or 48 hours (PROTECTON INTERIORWALL VK-500) at room temperature.

### Evaluation of phage inactivation by anti-viral materials

**Fig 2:**
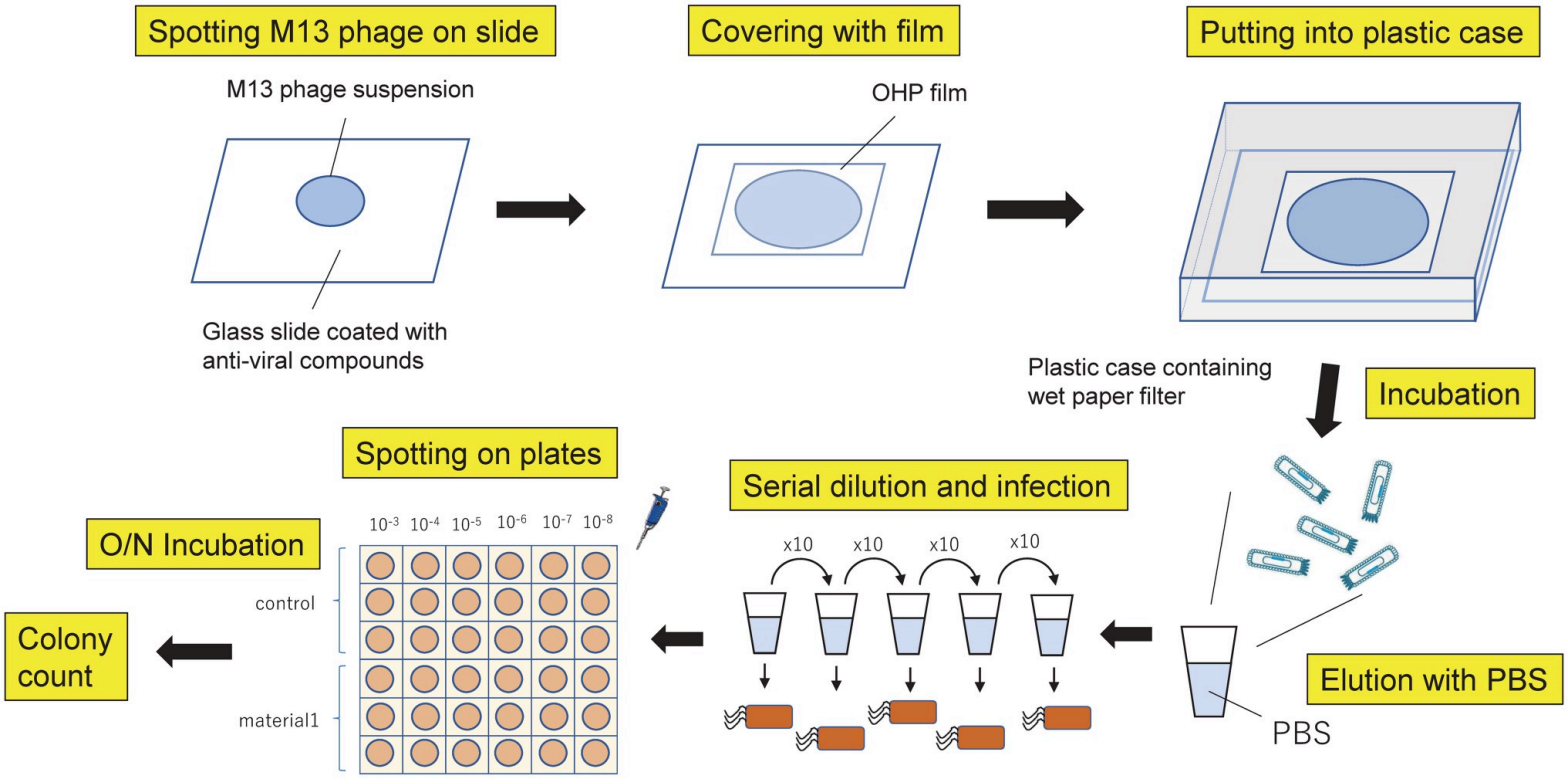
Schematic of the experiment using the M13 bacteriophage to evaluate the effects of antiviral materials.

Fig 2 shows a schematic of the experiment using the M13 bacteriophage. We inoculated 50 μL of the M13 phage suspension containing 1.4 x 10^11^ cfu phages in SM buffer onto glass slides (50 mm × 50 mm) coated with the antiviral reagents or a blank glass slide as the control and covered them with transparent polypropylene film (40 mm × 40 mm). After overnight incubation at room temperature under fluorescent lamp illumination (500 lx), we collected M13 phages by rinsing the glass slides with 5 ml of phosphate buffered saline and made a series of 1/10 dilutions. The eluted and serially diluted phages were mixed with *E*.*coli* strain XL-1 Blue cells at OD_600_ = 0.4–0.6 and incubated in water bath at 37°C for 30 min. Following incubation, 5 μl of each dilution were spotted onto grids on TYE plates containing ampicillin [19]. Following overnight incubation at 37°C, we counted the number of colonies in grids and calculated the number of active phages. For time course experiment, we collected M13 bacteriophage from the slides at each time point followed by making serial dilution and *E*.*coli* infection.

### Evaluation of influenza virus and Qβ phage inactivation by anti-viral materials

Antiviral effects of the materials against influenza virus and bacteriophage Qβ phage were evaluated by third parties following Industrial standards. As for influenza virus, virus suspension containing 1 × 10^7^ pfu/ml to 5 × 10^7^ pfu/ml in 0.4 ml of EMEM medium supplemented with kanamycin sulfate, termed as maintenance medium, were inoculated onto 50 mm × 50 mm sized test slides. The inoculated slides were covered with transparent film and incubated at 25 ± 1 ºC for 24 hours. Following incubation, the virus was collected with 10 ml of Soybean casein digest broth with lecithin and polyoxyethylene sorbitan monooleate broth (SCDLP broth). Ten-fold serial dilutions were prepared and used to infect MDCK cells followed by 1 hour incubation at 34 ºC to allow the virus to adsorb to the cells. Following wash of the wells with 3 ml of maintenance medium, 3 ml of the agar containing medium into the wells for plaque assay. After confirming the agar coagulates at room temperature, the plates were incubated at 34 ºC for 2 days to 3 days cells were stained with methylene blue solution and formed plaques were counted. As for Qβ phage, 0.15 ml of virus suspension containing 6.7 × 10^6^ pfu/ml to 2.6 × 10^7^ pfu/ml phages in 1/500 ordinary bouillon medium were inoculated onto 50 mm × 50 mm sized test slides. The inoculated slides were covered with transparent film and incubated at 25 ± 3 ºC under 500 lux light for 4 hours. Following incubation, the phage was collected with 10 ml of SCDLP broth. Ten-fold serial dilutions were prepared and used to infect *E*.*coli* cells of ATCC23631 strain. The infected *E*.*coli* cells were suspended in LB broth containing agar, which is warmed at 45 ± 1 ºC in water bath, and poured on agar plates. After confirming the agar coagulates at room temperature, the plates were incubated at 37 ± 1°C for 18 ± 2 hours and formed plaques were counted.

## Results

### Estimation of inactivation of the M13 phage by treatment with antiviral materials

To evaluate the screening system using bacteriophage M13 developed in this study, we assessed anti-viral effect of treatments with commercially available photocatalytic particle-based anti-viral reagents, PROTECTON INTERIORWALL VK-500 and PROTECTON BARRIERX Spray on M13 phage. The anti-viral effect of those anti-viral reagents were validated by officially approved third parties following Industrial Standards (JIS 1756R for Qβ phage and ISO 21702 for influenza virus) (S1 Fig).

Schematic of the experiment using M13 bacteriophage is shown in Fig 2. We inoculated M13 phage suspension onto glass slides coated with the anti-viral reagents or a blank glass slide as control, followed by covering with transparent films. After overnight incubation at room temperature in bright light, we collected M13 phage by rinsing the glass slides with PBS and made a series of 1/10 dilution. Subsequently, we infected *E*.*coli* cells with serially diluted M13 phage and spotted 5 μl of each *E*.*coli* cells into grids on TYE plates containing ampicillin. Following overnight incubation at 37 ºC, we counted the number of colonies in a grid.

**Fig 3:**
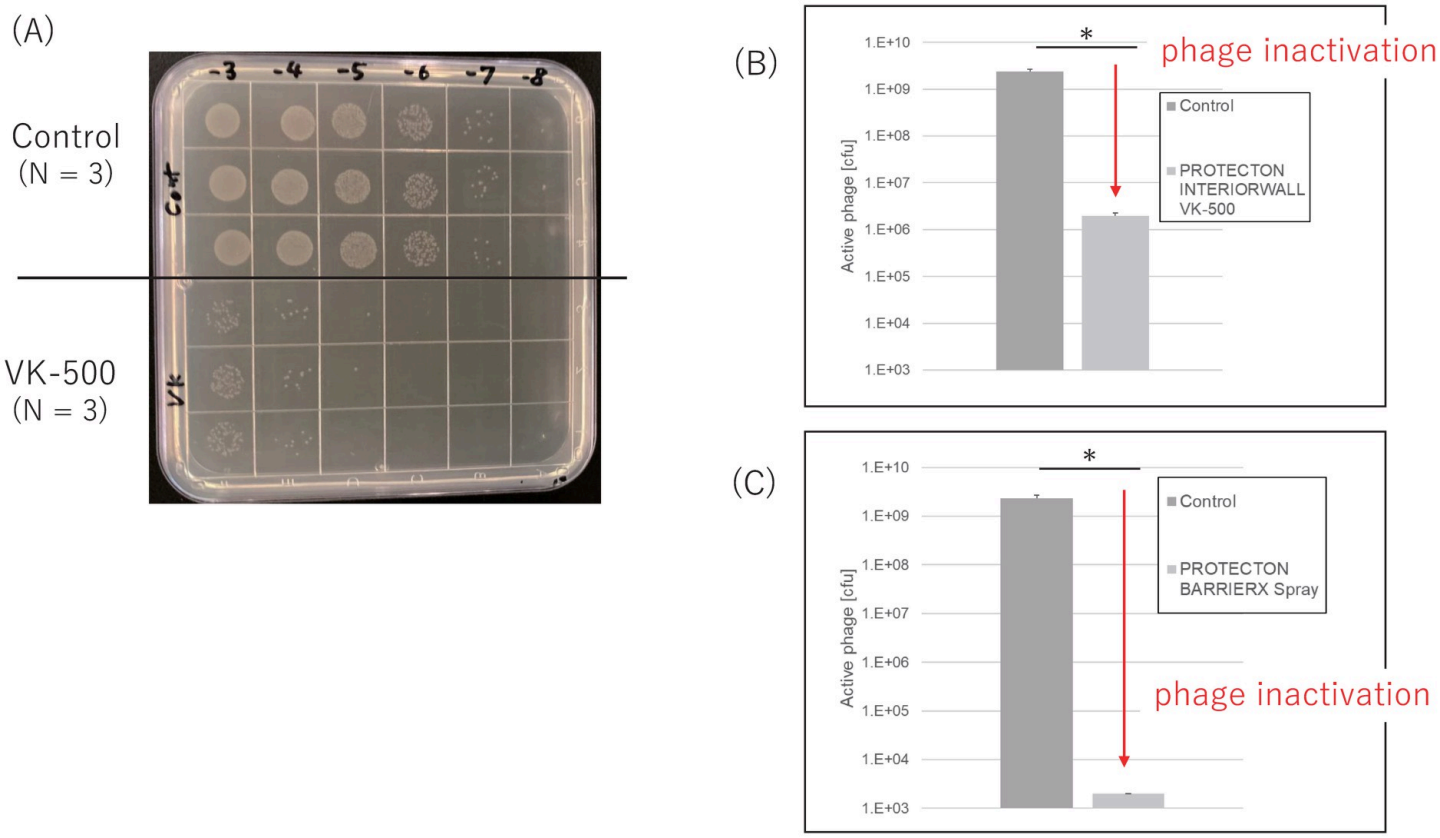
Validation of phage inactivation using commercially available antivirus products. (A) Representative picture of the TYE plate in which phage-infected *E*. *coli* cells were spotted. The dynamic range was set as 10^−3^ to 10^−8^ dilution in this experiment. (B, C) The calculated numbers of active phages after treatment with (B) PROTECTON INTERIORWALL VK-500 and (C) PROTECTON BARRIERX Spray. The experiments were repeated 3 times and student’s T-test were used as statistical analysis. p<0.001.

The number of colonies derived from M13 phage infection was drastically reduced by treatment with the antiviral reagents PROTECTON INTERIAL VK-500 and PROTECTON BARRIERX Spray (Fig 3A). We calculated the number of colony forming units (cfus) of M13 phages for each sample and then evaluated the degree of M13 phage inactivation via comparison with control samples. The cfu values were decreased by more than hundred-fold, indicating that more than 99% of M13 phages were inactivated (Fig 3B and 3C) These reductions agree with their antiviral effects on influenza virus and bacteriophage Qβ (S1 Fig), which validates the relevance of the novel method developed in this study to screen for antiviral materials.

**Fig 4:**
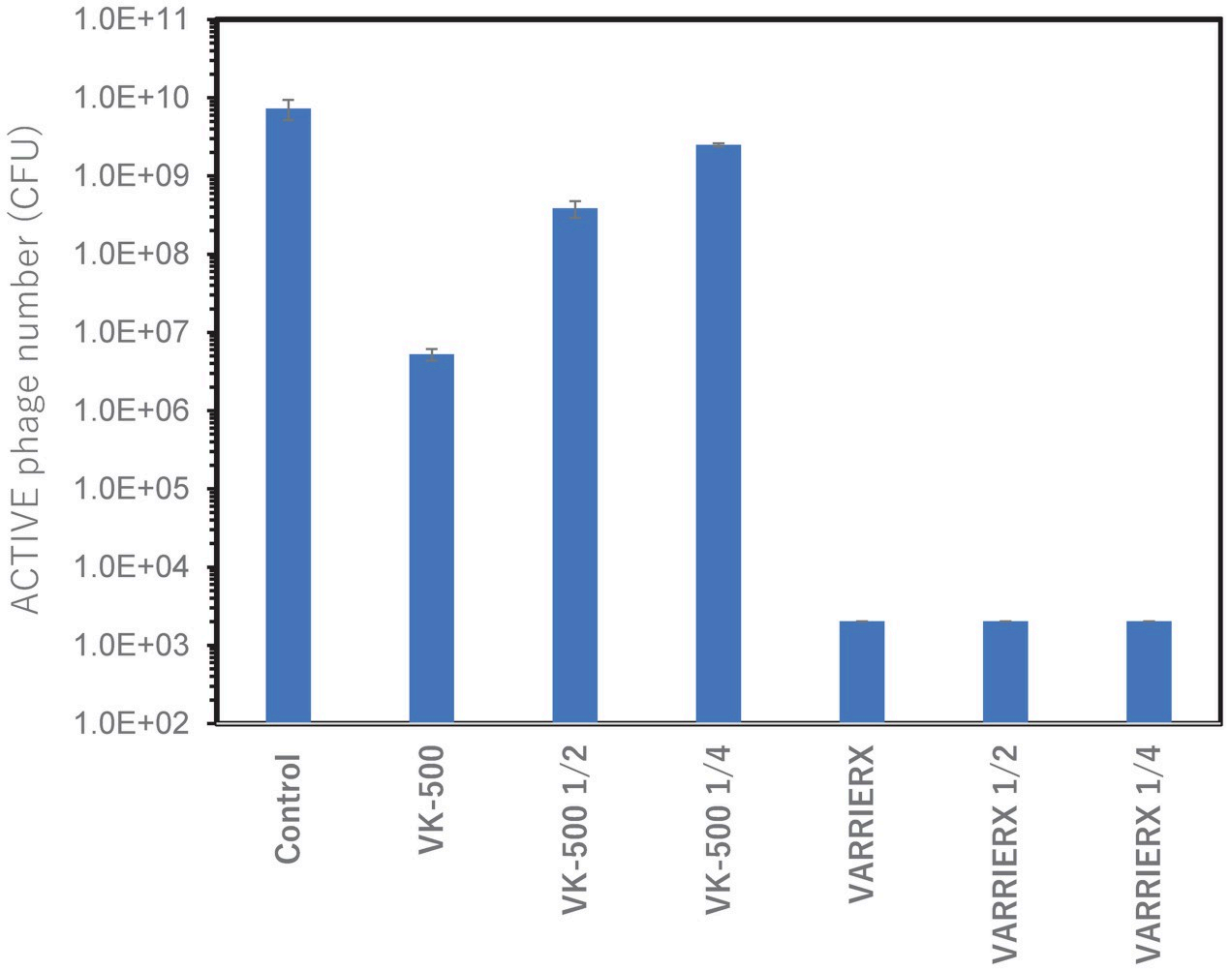
Effect of serially diluted anti-viral materials.

Subsequently, we evaluated the anti-viral effect of serially diluted each material. As shown in Fig 4, the effect of VK-500 was gradually diminished by serial dilution. In contrast, BARRIERX Spray showed strong anti-viral activity even with lower concentration. In this way, the effect of antiviral materials can be quantitatively evaluated by using our novel platform.

**Fig 5:**
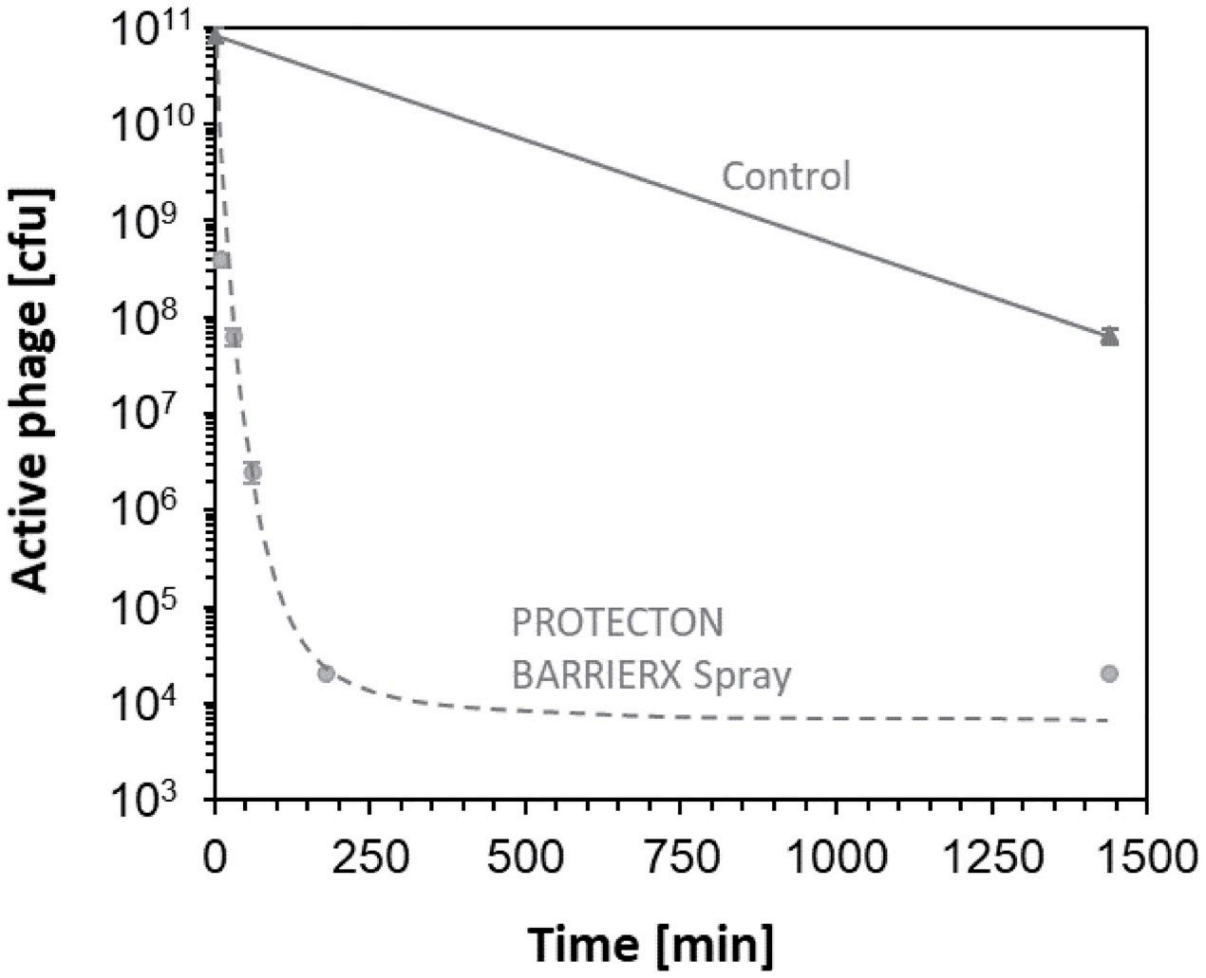
Time course experiment using PROTECTON BARRIERX Spray. Phages were collected at 10, 30, 60, 180, and 1440 min time points and active phage number was calculated.

We also evaluated the inactivation of M13 bacteriophages by PROTECTON BARRIERX Spray over time. The number of active phages gradually decreased over time (Fig 5), showing that the antiviral material inactivated the bacteriophages in a time-dependent manner.

## Discussion

In this study, we developed a novel screening method using the bacteriophage M13 to evaluate antiviral materials. Utilization of a phagemid, which provided *E*.*coli* cells with antibiotic resistance upon infection with M13 phages harboring the phagemid, enabled efficient screening. Because the number of cfus can be calculated from only a 5 μl spot of phage-infected *E*.*coli* cells in a grid, the platform enable evaluation of multiple conditions in a single plate at the same time, leading to the effective screening.

Previous studies have shown that different antimicrobial materials have distinct antiviral activities [11], likely due to differences in the composition of the viral surface. For example, Ly-Chatain et al. reported that the antiviral effect of cationic compounds on bacteriophages depended greatly on both the type of bacteriophages and the structure of antiviral compounds [11]. Therefore, it will be valuable to test the antiviral activity of novel antimicrobial materials against different kinds of viruses to develop materials that can protect against broad-spectrum viruses.

Importantly, as shown in Fig 5, the number of active phages were decreased by incubation on control glass slide. This result would indicate that M13 phage may be partially inactivated by some reaction with glass slide itself. Therefore, it is essential to prepare control glass slide without coating to evaluate antiviral activity of slide coating materials.

We used two types of antiviral materials, PROTECTON BARRIERX Spray and PROTECTON INTERIORWALL VK-500, as model materials. Since both materials contain TiO_2_-based photocatalytic particles, the antiviral effect was caused by photocatalytic reactions [8, 9]. Intriguingly, the two materials showed quite large difference in antiviral activity. This difference is thought to be derived from following 2 issues: presence of pigments and difference of film thickness. While PROTECTON BARRIERX Spray does not contain pigments, PROTECTON INTERIORWALL VK-500 contain pigments in the product. Also, film thickness of each coating was significantly different; several hundreds nm for PROTECTON BARRIERX Spray and several tens μm for PROTECTON INTERIORWALL VK-500, respectively. Since photocatalytic particles are considered to exert antiviral activity through direct contact with target viruses, these differences would be critical.

In this study, we used the filamentous phage M13, which has five kinds of coat proteins. Although this filamentous phage has not been used to evaluate antimicrobial effects, it has been widely used in phage display system in which the phage infection does not lead to cell lysis but provides resistance against antibiotics by introducing phagemid into infected bacterial cells. We took advantage of this system to develop an effective high-throughput system for screening potential antiviral materials. This new system enable more rapid screening than typical screening methods and thus should accelerate the development of novel antiviral materials.

## Supporting information

S1 Fig Validation of commercially available anti-virus products using traditional viruses. (A, B) Effect of PROTECTON VK-500 on Influenza and Qβ phage, respectively. (C, D) Effect of PROTECTON BARRIERX Spray on Influenza and Qβ phage, respectively.
(PDF)

S1 File Author contributions. (DOCX)

S1 Data (XLSX)

S2 Data (XLSX)

S3 Data (XLSX)

S4 Data (XLSX)
