# Supplementary material for: Development of a high-throughput method to screen novel antiviral materials
Source: PLoS One. 2022 Apr 27;17(4):e0266474. doi: 10.1371/journal.pone.0266474 (PMC9045606; doi:10.1371/journal.pone.0266474)
Supplement: S1 Fig — (A, B) Effect of PROTECTON VK-500 on Influenza and Qβ phage, respectively. (C, D) Effect of PROTECTON BARRIERX Spray on Influenza and Qβ phage, respectively. (PDF) [file pone.0266474.s001.pdf]

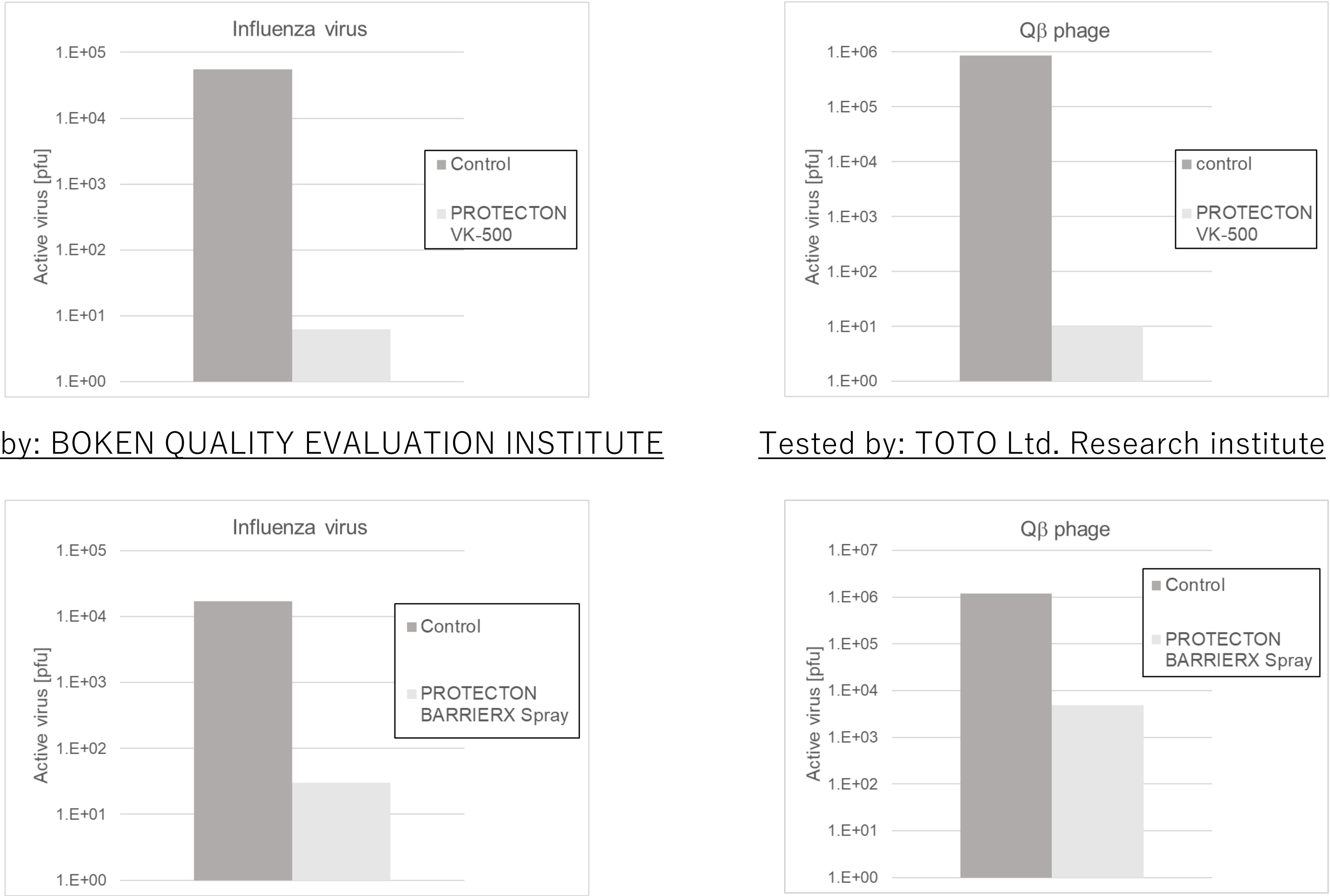

Tested by: BOKEN QUALITY EVALUATION INSTITUTE

Tested by: TOTO Ltd. Research institute

Tested by: Japan Food Research Labolatories (JFRL)

Tested by: TOTO Ltd. Research institute

Figure S1
