# Supplementary material for: Development of a high-throughput method to screen novel antiviral materials
Source: PLoS One. 2022 Apr 27;17(4):e0266474. doi: 10.1371/journal.pone.0266474 (PMC9045606; doi:10.1371/journal.pone.0266474)
Supplement: S1 File — (DOCX) [file pone.0266474.s002.docx]

**Author contributions**

**Conceptualization**: Makoto Nakakido, Kouhei Tsumoto

**Data Curation**: Makoto Nakakido, Naoki Tanaka, Ayako Shimojo, Nobuhiro Miyamae

**Formal Analysis**: Makoto Nakakido, Naoki Tanaka, Ayako Shimojo, Nobuhiro Miyamae

**Investigation**: Makoto Nakakido, Naoki Tanaka, Ayako Shimojo, Nobuhiro Miyamae

**Methodology**: Makoto Nakakido

**Project Administration**: Makoto Nakakido, Kouhei Tsumoto

**Resources**: Makoto Nakakido, Naoki Tanaka, Ayako Shimojo, Nobuhiro Miyamae, Kouhei Tsumoto

**Supervision**: Kouhei Tsumoto

**Visualization**: Makoto Nakakido, Naoki Tanaka, Ayako Shimojo, Nobuhiro Miyamae

**Writing – Original Draft Preparation**: Makoto Nakakido

**Writing – Review & Editing**: Naoki Tanaka, Ayako Shimojo, Nobuhiro Miyamae, Kouhei Tsumoto
